# Impaired chronotropic response to physical activities in heart failure patients

**DOI:** 10.1186/s12872-017-0571-9

**Published:** 2017-05-25

**Authors:** Hong Shen, Jianrong Zhao, Xiaohong Zhou, Jingbo Li, Qing Wan, Jing Huang, Hui Li, Liqun Wu, Shungang Yang, Ping Wang

**Affiliations:** 1 Department of Cardiology, Shanghai Sixth People’s Hospital, Shanghai Jiao Tong University, Shanghai, China; 2 Department of Cardiology, Ruijin Hospital Luwan Branch, Shanghai Jiao Tong University School of Medicine, Shanghai, China; 3 Cardiac Rhythm and Heart Failure, Research and Technology, Medtronic plc, MV, Minneapolis, MN USA; 4 Department of Cardiology, Ruijin Hospital, Shanghai Jiao Tong University School of Medicine, Shanghai, China; 5 Medtronic Shanghai Innovation Center, Medtronic (Shanghai) Ltd., Shanghai, China

**Keywords:** Chronotropic incompetence, Heart rate, Heart failure rehabilitation, Treadmill exercise testing, 6 min hall walk

## Abstract

**Background:**

While exercise-based cardiac rehabilitation has a beneficial effect on heart failure hospitalization and mortality, it is limited by the presence of chronotropic incompetence (CI) in some patients. This study explored the feasibility of using wearable devices to assess impaired chronotropic response in heart failure patients.

**Methods:**

Forty patients with heart failure (left ventricular ejection fraction, LVEF: 44.6 ± 5.8; age: 54.4 ± 11.7) received ECG Holter and accelerometer to monitor heart rate (HR) and physical activities during symptom-limited treadmill exercise testing, 6-min hall walk (6MHW), and 24-h daily living. CI was defined as maximal HR during peak exercise testing failing to reach 70% of age-predicted maximal HR (APMHR, 220 – age). The correlation between HR and physical activities in Holter-accelerometer recording was analyzed.

**Results:**

Of 40 enrolled patients, 26 were able to perform treadmill exercise testing. Based on exercise test reports, 13 (50%) of 26 patients did not achieve at least 70% of APMHR (CI patients). CI patients achieved a lower % APMHR (62.0 ± 6.3%) than non-CI patients who achieved 72.0 ± 1.2% of APMHR (*P* < 0.0001). When Holter-accelerometer recording was used to assess chronotropic response, the percent APMHR achieved during 6MHW and physical activities was significantly lower in CI patients than in non-CI patients. CI patients had a significantly shorter 6MHW distance and less physical activity intensity than non-CI patients.

**Conclusion:**

The study found impaired chronotropic response in 50% of heart failure patients who took treadmill exercise testing. The wearable Holter-accelerometer recording could help to identify impaired chronotropic response to physical activities in heart failure patients.

**Trial registration:**

ClinicalTrials.gov ID NCT02358603. Registered 16 May 2014.

## Background

Heart failure patients experience a variety of symptoms, of which the most frequently presenting symptoms are exertional breathlessness, fatigue, and intolerance to physical activities, leading to poor quality of life. While, historically, patients with heart failure were suggested to avoid exertion, exercise-based cardiac rehabilitation has now been recognized as having a beneficial effect on heart failure hospitalization and mortality [1–4]. Currently, exercise prescription is commonly based on heart rate. However, exercise level or target intensity that depends on achievable maximal heart rate can be significantly limited in heart failure patients with chronotropic incompetence (CI) [3, 4].

In response to physical exercise that increases oxygen demand, heart rate (HR), via enhanced sympathetic activity and/or withdrawal of parasympathetic activity, increases to meet the body’s metabolic demand. CI is a condition when HR increases inadequately in response to increased metabolic demand [5–8]. Patients with CI often show exercise intolerance and hence have impaired quality of life. Furthermore, studies have also demonstrated that CI is an independent risk factor for major cardiovascular adverse events including overall mortality [9–12]. The prevalence of CI in patients with heart failure has been reported to vary from 25% to 65% [8, 9, 13, 14].

At present, the method that clinically assesses CI is dynamic incremental exercise testing or exercise tolerance testing during which cardiac chronotropic capacity is measured [6–12]. CI is believed to be present if maximal HR during the peak of exercise testing cannot reach a certain percentage of the age-predicted maximal heart rate value (APMHR, usually 220 – age), such as 80%, or even 70% [6–8, 12]. The magnitude of the change in HR during exercise testing is also used to detect impaired chronotropic incompetence, e.g., the HR reserve (the difference between maximal HR during peak exercise and resting HR prior to exercise testing) [8, 9, 11, 12].

While exercise-based cardiac rehabilitation has been recommended for heart failure patients, the adoption of the guidelines is negatively impacted by CI. In other words, if CI is assessed and better managed, target intensity and exercise formula can be better prescribed. The purpose of the present study was to investigate a method of heart rate and physical activity recording to assess chronotropic response during treadmill exercise testing and daily physical activities in patients with heart failure.

## Methods

The study was conducted in two centers and the study protocol was approved by both hospitals’ Institutional Review Boards and in compliance with the Declaration of Helsinki. All patients completed written informed consent.

### Selection of patients

Forty patients were enrolled between June 2014 and April 2015. The inclusion criteria included: (1) patients had heart failure based on ESC 2012 heart failure guidelines; (2) patients’ NYHA classification was ranged from I to III and left ventricular ejection fraction (LVEF) was more than 35%; (3) patients were capable of performing moderate exercise. Patients were excluded if they (1) received a pacemaker, implantable cardioverter-defibrillator or cardiac resynchronization therapy device; (2) had persistent or permanent atrial fibrillation; (3) were incapable of exercise due to angina, heart failure decompensation, ST deviation >2 mm, or active pericarditis and myocarditis; (4) had acute myocardial infarction <45 days; (5) had uncontrolled hypertension; or (6) had other medical issues that would compound the present study.

### Study procedures

All enrolled patients received 24-h Holter ECG (NorthEast Monitoring, Inc., Maynard, MA, USA) for HR and ActiGraph GT3 accelerometer (ActiGraph, Shalimer, FL, USA) monitoring for determining the magnitude of physical activities. The clinically validated Actigraph accelerometer determined counts of 10-s acceleration activities in X-, Y-, and Z- vectors with each count equal to 16 m-g per second where the g is 9.825 m•s^−2^ [15–17]. The vector magnitude (VM) was calculated as the square root of the sum of the second power of X-, Y-, and Z-vector counts. Once the Holter-Actigraph monitoring was in place, patients underwent a 6 min hall walk (6MHW) and a symptom-limited treadmill exercise test. Some patients, based on physicians’ clinical judgment and patients’ willingness, did not participate in treadmill exercise testing. All enrolled patients received stable medication and there was no requirement to withhold any medication. Patients’ demographic information, echocardiography, blood pro-NT-BNP, and medications were collected at enrollment.

The symptom-limited maximal treadmill exercise test adopted the modified Naughton-Balke method [18]. Briefly, the total time of treadmill exercise testing was 14 min with two minutes at base speed with slope 0 (no inclination), two minutes at slope 1 level and speed 2, and two minutes at each of next five slopes with no change in speed. During exercise testing, 12-lead electrocardiogram and blood pressure were recorded in addition to Holter-Actigraph recording.

### Assessment of chronotropic incompetence

The assessment of chronotropic incompetence was based on the achieved maximal HR during peak treadmill exercise. CI is defined as maximal HR at peak treadmill exercise that fails to reach an arbitrary percentage of APMHR, for which 70% was chosen as the criterion for diagnosis of CI in heart failure patients [12, 19, 20]. In the present study, as most patients took beta-blockers, a newly proposed equation based on HR reserve for determining chronotropic impairment was also used to assess CI, e.g., chronotropic index for patients with beta blockers (called chronotropic index-β) = (HR at peak exercise – resting HR) / (119 + (resting HR / 2) – (age / 2) – resting HR) [11].

Chronotropic response during daily living was assessed by 24-h Holter-Actigraph recording. An event of physical activity was defined once Actigraph recording reported 10 or more than 10 s of activities. Maximal Actigraph VM value in counts and HR were determined for each physical activity. Resting HR was determined by averaging HR values in the time window from 10 min after the previous activity event to the time right before the incoming activity. Thus, for each physical activity, three parameters, e.g., resting HR, maximal HR and maximal Actigraph VM, were obtained. The regression between HR values and corresponding Actigraph activity levels was performed for 24-h daily living. The overall intensity of daily physical activities was expressed as the averaged maximal VM of all physical activities during the recording window.

### Statistical analysis

Continuous variables are expressed with mean ± standard deviation. The categorical variables are used for number of patients. Comparisons for continuous variables were made using *t* test whenever appropriate. Categorical variables were compared using the Chi square test. The correlation between the CI values by exercise test reports and those by Holter-Actigraph recording was determined using Pearson correlation coefficients with corresponding correlation equations. The simple linear regression between HR and physical activities for 24-h Holter-Actigraph recording was performed. To minimize the impact of variations among individuals on regression analysis results, HR was taken as a function of the percentage of the maximal daily physical activity. Binary logistic regression was used to detect the effect of variables in patient characteristics (age, weight, gender, coronary heart disease, cardiomyopathy, LVEF, NYHA, β-blockers) on the results (CI or no-CI) of treadmill exercise testing. A two-tailed *P* value of ≤0.05 was considered significant. The statistical tool SPSS was used for the statistical analyses.

## Results

### Treadmill exercise testing

All 40 enrolled patients (median age: 57.5 years old, range 30–70) were diagnosed with heart dysfunction with LVEF 44.6 ± 5.8% and symptoms of exertion-associated dyspnea, asthenia and fatigue. Most patients (60%) were in NYHA class II. The clinical characteristics of patients are presented in Table 1. Of the 40 enrolled patients, 26 patients underwent treadmill exercise testing and the remaining 14 patients declined treadmill exercise testing. There was no significant difference in clinical characteristics between patients taking treadmill exercise testing and those not taking (Table 2). In 13 of 26 patients (50%) who underwent treadmill exercise testing, maximal heart rate at peak exercise did not reach at least 70% of APMHR (CI patients) while in the remaining 13 patients (50%) maximal heart rate did reach ≥70% of APMHR (non-CI patients). Of 5 patients with NYHA III, maximal heart rate in 2 patients reached ≥70% of APMHR but not in other 3 patients. The binary logistic regression analysis showed only gender affected the CI results of treadmill exercise testing with an odd ratio 0.04 (for male), 0.002–0.805 95% CI and *P* value = 0.036.

**Table 1:** Patient characteristics and results of treadmill exercise testing

	All patients (*N* = 40)	≥70% APMHR (*N* = 13)	<70% APMHR (*N* = 13)	*P* value
Age (years)	54.4 ± 11.7	53.1 ± 10.9	52.5 ± 13.8	0.905
Weight (Kg)	74.8 ± 17.6	78.2 ± 19.3	74.4 ± 19.4	0.634
Male, N (%)	30 (75)	13 (92.3)	7 (53.8)	0.005
NYHA class	2.2 ± 0.6	1.9 ± 0.6	2.2 ± 0.4	0.166
LVEF (%)	44.6 ± 5.8	45.8 ± 5.5	42.0 ± 5.2	0.090
LVESD (mm)	45.9 ± 7.6	45.2 ± 7.1	49.9 ± 4.8	0.066
LVEDD (mm)	59.4 ± 7.7	58.2 ± 7.8	62.8 ± 4.5	0.084
6MHW (m)	413.8 ± 86.9	486.2 ± 62.0	416.7 ± 37.1	0.003
SBP (mmHg)	125.6 ± 18.1	126.6 ± 20.1	126.8 ± 17.4	0.987
DBP (mmHg)	76.8 ± 9.9	79.2 ± 10.0	76.2 ± 11.4	0.528
BNP (pg/mL)	147.5 ± 225.8	114.7 ± 96.8	124.0 ± 145.9	0.855
CAD, N (%)	15 (37.5)	4 (33.3)	5 (35.7)	0.680
Cardiomyopathy, N (%)	35 (87.5)	11 (84.6)	12 (92.3)	0.539
Β-blocker, N (%)	38 (95.0)	12 (92.3)	13 (100.0)	0.308
ACE inhibitor, N (%)	16 (40.0)	6 (46.2)	8 (61.5)	0.431
Diuretics, N (%)	20 (50.0)	7 (53.8)	7 (53.8)	1.000
Digoxin, N (%)	10 (25.0)	2 (15.4)	6 (46.2)	0.089
Patients taking treadmill exercise testing (*N* = 26)				
Rest HR (bpm)	78.2 ± 10.9	80.1 ± 10.1	76.2 ± 11.8	0.373
Maximum HR (bpm)	112.0 ± 14.8	120.1 ± 8.8	103.9 ± 15.4	0.003
HR reserve (bpm)	33.9 ± 11.9	40.0 ± 10.5	27.2 ± 10.2	0.006
APMHR	167.6 ± 12.5	166.8 ± 11.4	168.4 ± 14.0	0.775
Achieved %APMHR	67.0 ± 6.8	72.0 ± 1.2	62.0 ± 6.3	<0.0001
C index-β (%)	62.7 ± 19.6	75.3 ± 12.9	46.4 ± 15.3	<0.0001

**Table 2:** Baseline characteristics in patients with or without treadmill exercise testing

	TET (*N* = 26)	No TET (*N* = 14)	*P* value
Age (years)	52.8 ± 12.4	57.5 ± 9.4	0.233
Weight (Kg)	76.3 ± 19.4	72.0 ± 13.0	0.471
Male, N (%)	20 (76.9)	10 (71.4)	0.702
LVEF (%)	43.9 ± 5.7	45.8 ± 5.9	0.346
NYHA class	2.1 ± 0.5	2.3 ± 0.7	0.318
LVESD (mm)	47.5 ± 6.5	42.8 ± 8.5	0.062
LVEDD (mm)	60.5 ± 6.8	57.2 ± 8.8	0.208
6MHW (m)	451.4 ± 61.8	344.1 ± 83.9	<0.001
BNP (pg/mL)	119.4 ± 123.8	119.8 ± 336.1	0.294
CAD, *N* (%)	9 (34.6)	5 (35.7)	0.945
Cardiomyopathy, *N* (%)	23 (88.5)	12 (85.7)	0.802
Β-blocker, *N* (%)	25 (96.2%)	13 (92.9)	0.648

*TET* treadmill exercise testing

When β-blocker use was taken into account in the analysis using chronotropic index-β, 38.5% patients (10 / 26) failed to achieve 60% chronotropic index-β. While there were no significant differences in age and corresponding APMHR between CI and non-CI patients, CI patients achieved significantly lower maximal HR, HR reserve, and chronotropic index-β than non-CI patients (Table 1).

**Fig. 1:**
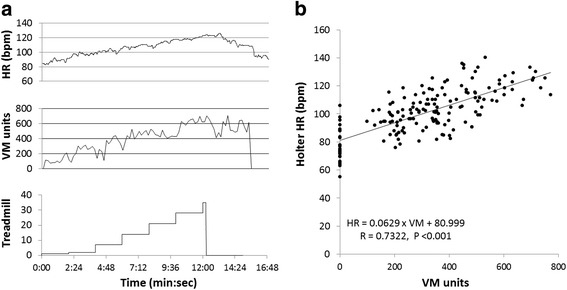
Heart rate and Actigraph VM recording during treadmill exercise testing. **a** Holter heart rate (*top*) and Actigraph activity in VM (*middle*) recording during 7 levels of treadmill exercise testing (*bottom*) in one patient. **b** the correlation between Holter heart rate and levels of VM during treadmill exercise testing in all 26 patients. Each dot represents the measurement of a maximal VM value in one of 7 levels of treadmill exercise testing

Figure 1a shows an example of HR and physical activity levels recorded by the Holter-Actigraph devices during treadmill exercise testing in a patient. There was a significant correlation between HR and activity levels, e.g., HR = (0.0629 × VM) + 80.999 (*R* = 0.7322, *P* < 0.0001, Fig. 1b), demonstrating an increase in HR in response to the increase in physical activity levels. The magnitude of physical activity level (VM) during peak exercise appeared lower in CI patients (VM: 380.3 ± 194.5 unit counts) than in non-CI patients (VM: 475.5 ± 142.0 unit counts, *P* = 0.183 vs. the CI patients).

### HR analysis during 24-h daily living

**Fig. 2:**
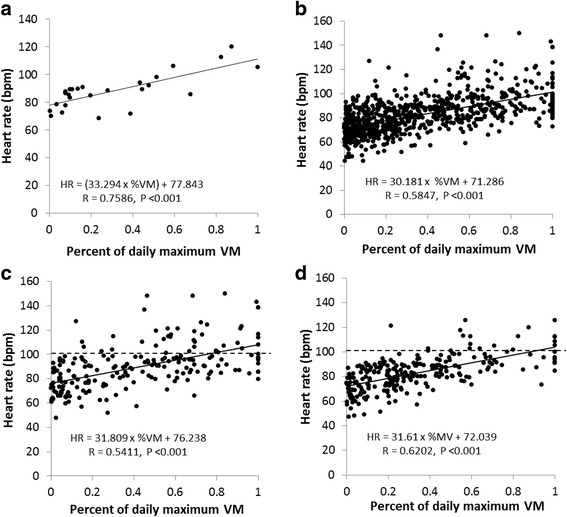
Correlation between heart rate and physical activities during daily living. **a** the correlation between heart rate (bpm, the ordinate) and the percentage of each maximal daily physical activity over the greatest maximal daily physical activity (the abscissa) in one patient; **b** the correlation for all 40 patients; **c** the correlation for 13 non-CI patients; **d** the correlation for 13 CI patients. Dashed line in (**c** and **d**) represents the maximal HR above 100 bpm during physical activity events

An example of the relationship between HR and the VM of physical activities from a patient is presented in Fig. 2a, showing chronotropic response. The overall relationship between HR and all physical activities over the 24-h time window in all patients is presented in Fig. 2b, in which HR is significantly correlated with VM levels of physical activities. The correlation coefficients were at the same level between non-CI patients (Fig. 2c) and CI patients (Fig. 2d). However, the frequency of HR over 100 bpm during activity events was significantly higher (*P* = 0.0006) in non-CI patients (20% of all activity events, Fig. 2c) than in CI patients (8.8% of all activity events, Fig. 2d).

Maximal HR achieved during daily physical activities was 106.7 ± 11.8 bpm (equal to 63.9 ± 6.8% APMHR) in CI patients, which was lower than 121.8 ± 17.3 bpm (73.0 ± 9.6% APMHR) in non-CI patients (*P* = 0.020 vs. CI patients). The maximal HR reserve achieved during the 24-h window was 31.9 ± 7.0 bpm in CI patients, significantly smaller than that in non-CI patients (40.9 ± 12.2 bpm *P* = 0.035 vs. CI patients). The overall intensity (VM) of daily maximal physical activities was 333.2 ± 87.6 counts in CI patients, which was smaller than in non-CI patients (390.0 ± 118.8 counts, *P* = 0.178 vs. CI patients).

### HR analysis during 6-min hall walk

Maximal HR achieved during 6MHW (100.1 ± 19.6 bpm) was significantly lower than maximal HR achieved during the peak of treadmill exercise testing (112.0 ± 14.8 bpm, *P* = 0.012). However, there was a significant correlation between the 6MHW distance and the corresponding percent APMHR achieved during treadmill exercise testing (Distance = 571.7 × %APMHR + 68.6, *R* = 0.615, *P* = 0.001). The percent APMHR achieved during 6MHW was 57.5 ± 6.3% in CI patients which was significantly smaller than that in non-CI patients (63.5 ± 6.9%, *P* = 0.038 vs. CI patients) and the 6MHW distance was significantly shorter in CI patients (416.7 ± 37.1 m) than in non-CI patients (486.2 ± 62.0 m, *P* = 0.003, Table 1).

### CI assessment in patients without treadmill exercise testing

Fourteen patients did not take treadmill exercise testing based on patients’ willingness and physicians’ clinical judgment. In these 14 patients, the achieved percent APMHR was 60 ± 6.3% during daily physical activities and 56.4 ± 7.9% during 6MHW, both values of the percent APMHR were significantly lower than in non-CI patients (73.0 ± 9.6% APMHR during daily activities and 63.5 ± 6.9% during 6MHW, both *P* values <0.05 vs. corresponding values in 14 patients), but comparable to the values of the percent APMHR in CI patients (63.9 ± 6.8% APMHR during daily activities and 57.5 ± 6.3% during 6MHW, both *P* values >0.05 vs. the corresponding values in 14 patients). Moreover, the overall intensity (VM) of daily maximal physical activities was 315.5 ± 107.8 counts in VM, which appeared lower than in non-CI patients (390.0 ± 118.8 counts, *P* = 0.101) but not significantly different compared with CI patients (333.2 ± 87.6 counts, *P* > 0.05).

## Discussion

The present study used two methods to assess impaired chronotropic response to physical activities in patients with heart failure. The method of traditional treadmill exercise testing identified 50% of patients whose maximal HR during peak exercise failed to reach at least 70% of APMHR, a criterion for CI diagnosis in heart failure patients [12, 19, 20]. Holter-Actigraph recording and corresponding measurements showed a high concordance with the results of exercise testing. Furthermore, in the 24-h recording of HR and physical activities by the Holter-Actigraph system, the assessment of chronotropic response during 6-MHW and daily living found worse chronotropic response in CI patients than in non-CI patients, demonstrating the feasibility of assessment of impaired chronotropic response in heart failure patients by this method.

In the present study, maximal HR during peak exercise failed to reach at least 70% of APMHR in 50% of tested patients, thus CI was diagnosed accordingly for these patients. When HR reserve and chronotropic index-β were used to assess CI, 21 (80.8%) of 26 patients who took exercise testing failed to attain ≥80% of HR reserve and 10 (38.5%) patients failed to attain ≥60% of the chronotropic index-β. Thus, the CI incidence in heart failure population depends on the methods used for diagnosis [8]. The criterion of 70% APMHR was used in the present study, based on which the study found that (1) patients who met the CI diagnosis criterion had a significantly lower HR reserve and chronotropic index-β than those who did not meet the CI diagnosis criterion, and (2) HR changes in response to daily physical activities and the 6MHW distance were significantly smaller in patients who met the CI criterion than those who did not. When compared to the general population who have no heart failure and can reach the target heart rate during exercise testing (e.g., ≥85% APMHR) [7, 8], the heart failure patients in the present study whose heart rate during exercise testing could reach ≥70% APMHR, but still less than 85% might have reduced chronotropic response even though the CI could not be definitively diagnosed.

The present study applied a wearable recording system (Holter-Actigraph) during treadmill exercise testing in heart failure patients and assessed the correlation between treadmill exercise test results and measurements by Holter-Actigraph recording. The rationale to adopt Holter-Actigraph recording was to validate an ease-of-use tool that can be used to screen for and assess CI without the need of special infrastructure like a treadmill exercise test laboratory. The uniqueness of the present study was to use Holter-Actigraph recording to determine HR during physical activities during daily living including 6MHW and thus evaluate chronotropic capacity. The analysis of HR and physical activities based on Holter-Actigraph recording revealed a significant correlation between heart rate and physical activity levels, e.g., chronotropic response. Furthermore, the study found a significant correlation between the 6MHW distance and the percent APMHR achieved during exercise testing and reduced physical activity intensity in patients with impaired chronotropic incompetence. Thus, the Holter-Actigraph system can potentially be used to screen for CI and assess impaired chronotropic response in heart failure patients.

Exercise intolerance and symptoms of dyspnea and fatigue on effort are clinical manifestations in patients with heart failure. It is assumed that the appearance of these symptoms is more likely due to heart failure if rapid heart rate occurs in response to a moderate activity rather than the cause of CI in which there is a lack of a significant increase in heart rate. Thus, heart rate changes in response to a physical activity can be used to distinguish whether symptoms are caused by CI or not.

### Clinical perspectives

Recent studies have demonstrated a high incidence of CI in the heart failure population and CI has been recognized as an independent risk factor for cardiovascular morbidity and mortality. Daily exercise is recommended for patients with chronic heart failure. However, the exercise intensity in terms of magnitude and time interval still remains uncertain and the intensity of exercise is significantly influenced by the capability of chronotropic response. Furthermore, use of β-blockers complicates chronotropic response, leading to a reduced chronotropic response [8, 9, 11, 21–23]. On the other hand, β-blockers have been a standard therapy that prolongs survival of heart failure patients in several clinical trials [24–26]. There are approximately 5.8 million patients with heart failure in the United States and 23 million worldwide [27]. It can be assumed that the majority of heart failure patients would not receive an assessment of their chronotropic response. As shown in the present study, of 40 heart failure patients, 14 (35%) patients who declined treadmill exercise testing did have low %APMHR achieved during 6MHW and daily physical activities. Thus, if a simple, ease-of-use tool can be used to screen for CI and assess impaired chronotropic response in heart failure population, more patients with impaired chronotropic response could be identified and better managed during heart failure treatment including exercise-based cardiac rehabilitation and treatment with β-blockers. Rate-responsive pacing is an effective method to relieve symptoms caused by CI [8, 28]. However, such a therapy may be underutilized in the heart failure population because exercise intolerance-related symptoms are more often recognized as heart failure symptoms instead CI-caused symptoms. Exertional dyspnea and weakness-asthenia are common symptoms in both chronic heart failure and CI. If drug therapy, such as loop diuretics, does not effectively relieve exertion-related symptoms in heart failure patients with CI, rate-responsive pacing can be considered. In addition, CI can be a manifestation of sick sinus dysfunction whose symptoms include dyspnea, asthenia, fatigue, frequent dizziness, and possible fainting or syncope with the potential of precipitating or aggravating a state of heart failure [29]. Thus, rate-responsive pacing can be used if these patients receive a pacemaker. The present study clinically not only confirmed a high CI incidence in heart failure patients, but also investigated the feasibility of using Holter-Actigraph recording as an alternate and simpler tool to identify heart failure patients with CI and impaired chronotropic response. Prospective studies are needed to evaluate clinical benefits that can be provided by the assessment of chronotropic impairment with the use of tools as the Holter-Actigraph recording.

### Limitations

The present study used the symptom-limited maximal exercise test that did not have respiratory monitoring or measurement of peak oxygen consumption. Thus, the present study did not incorporate the information of metabolic and oxygen demand during exercise. Second, the present study had a relatively small sample size and enrolled heart failure patients with LVEF ranging from 35% to 53%. Moreover, exercise intolerance is one of the clinical manifestations of heart failure and use of β-blockers further complicates chronotropic response [8, 11, 21–23]. Thus, the variables for determining CI or chronotropic impairment derived from the present study need to be further confirmed in a clinical study with a large population including patients with all levels of LVEF and different medications.

## Conclusions

The present study found 50% of tested patients whose maximal HR during peak exercise failed to reach at least 70% of APMHR, a criterion for CI diagnosis in heart failure patients. Use of the HR-activity recording system identified worse chronotropic response and lower physical activity intensity in CI patients than in non-CI patients, demonstrating the feasibility of assessment of impaired chronotropic response in heart failure patients by this simple method. Clinical utility of non-invasive monitoring tools as the Holter-Actigraph system in diagnosing and treating CI should be investigated in a larger heart failure population.

## Abbreviations

6MHW: 6 min hall walk
APMHR: Age-predicted maximal heart rate value
CI: Chronotropic incompetence
ECG: Electrocardiography
HR: Heart rate
LVEF: Left ventricular ejection fraction
VM: Vector magnitude
